# Pulmonary Vein Isolation Followed by Biatrial Ablation of Rotational Activity in Patients with Persistent Atrial Fibrillation: Results of the Cryo-Vest Study

**DOI:** 10.3390/jcm13041118

**Published:** 2024-02-16

**Authors:** Kay Felix Weipert, Julie Hutter, Malte Kuniss, Patrick Kahle, Joerg Yogarajah, Andreas Hain, Johannes Sperzel, Alexander Berkowitsch, Christian W. Hamm, Thomas Neumann

**Affiliations:** 1 Department of Cardiology, Kerckhoff Heart Center, 61231 Bad Nauheim, Germany; j.hutter@kerckhoff-klinik.de (J.H.); m.kuniss@kerckhoff-klinik.de (M.K.); p.kahle@kerckhoff-klinik.de (P.K.); j.yogarajah@kerckhoff-klinik.de (J.Y.); a.hain@kerckhoff-klinik.de (A.H.); j.sperzel@kerckhoff-klinik.de (J.S.); a.berkowitsch@kerckhoff-klinik.de (A.B.); c.hamm@innere.med.uni-giessen.de (C.W.H.); t.neumann@kerckhoff-klinik.de (T.N.); 2 German Center for Cardiovascular Research (DZHK), Rhein-Main Partner Site, 61231 Bad Nauheim, Germany

**Keywords:** persistent atrial fibrillation, noninvasive mapping, ablation, cryoballoon, pulmonary vein isolation, rotational activity

## Abstract

**Background and Aims**: Noninvasive mapping allows the identification of patient-specific atrial rotational activity (RA) that might play a key role in the perpetuation of persistent atrial fibrillation (PsAF). So far, the impact of pulmonary vein isolation by cryoballoon (Cryo-PVI) on RA is unclear. Moreover, the long-term effect of periprocedural termination of AF during the ablation procedure is controversial. **Methods**: Noninvasive electrocardiographic mapping with a 252-electrode vest was performed in 42 patients with PsAF. After the first analysis, Cryo-PVI was performed. The RA was analyzed again and then targeted by radiofrequency catheter ablation. The primary clinical endpoint was periprocedural termination of AF. The secondary endpoint was freedom from any atrial arrhythmia >30 s during a 12-month follow-up. **Results**: In 33 patients (79%), right atrial RA was identified leading to biatrial ablation, and nine patients (21%) had left atrial RA only. Twelve patients (28.6%) converted from AF to sinus rhythm (SR) (Group A). Thirteen patients (30.9%) converted to atrial tachycardia (AT) (Group B). In 17 patients (40.5%), AF was not terminated by ablation (Group C). After a mean follow-up time of 13.8 months, 26 patients were free from AF and AT (61.9%). In terms of rhythm, control Group A (75%) and B (83.3%) showed higher success rates than Group C (33.3%) (*p* < 0.01). Cryo-PVI had no substantial impact on RA. **Conclusions**: The RA-based ablation approach showed acceptable success rates. Periprocedural termination of AF had a positive predictive impact on the outcome. No difference was observed between conversion to SR or to AT. Cryo-PVI had no impact on RA.

## 1. Introduction

Atrial fibrillation (AF) represents the arrhythmia with the highest incidence and prevalence worldwide, and is associated with a high rate of morbidity and mortality [1,2]. Moreover, AF affects a vast number of patients symptomatically in terms of fatigue, dyspnea, and palpitations. Patient selection is pivotal in aiming for sustained rhythm control management. Although catheter ablation of AF has evolved from being an investigational procedure to the most effective treatment option for symptomatic patients, the current guidelines treat catheter ablation as a second-line therapy with a Class IIa recommendation [3]. Notably, in contrast to ablation, long-term therapy with antiarrhythmic drugs (AAD) has been shown to be associated with considerable cardiac and extracardiac side effects. While patients with paroxysmal AF (PAF) have encouraging clinical outcomes, with success rates of approximately 80% after possibly multiprocedural pulmonary vein isolation (PVI) alone after five years and 60% after 10 years, rhythm control in patients with persistent atrial fibrillation (PsAF) seems to be far more difficult to maintain [4,5,6,7,8]. After a single catheter ablation approach, rhythm control in patients with PsAF is as low as 25% after a six-year follow-up period [9].

Different approaches to the treatment of PsAF have evolved, including the creation of linear lesions and/or targeting low-voltage areas and complex fractionated electrogram (CFAE) ablation, with modest success rates. Freedom from AF recurrence is slightly higher for minimally invasive surgical ablation approaches, but is also associated with more severe procedural adverse events [10]. All of these methods lack specificity and therefore can lead to extensive and conceivably unnecessary ablation, which carries a potentially increased periprocedural risk, may produce new pro-arrhythmic lesions, and may have additional deleterious effects on the atrial hemodynamic function.

New technologies have allowed the detection of patient-specific atrial rotational activity that might play a key role in the perpetuation of PsAF. While endocardial mapping systems, such as focal impulse and rotor modulation (FIRM), have shown inconsistent results, a unique noninvasive body surface mapping technique has shown promising effects in treating patients with PsAF [11,12,13,14,15]. The present investigation is the first to compare biatrial rotational activity before and after pulmonary vein isolation (PVI). To our knowledge, this is also the first study to combine PVI using cryoballoon technology (cryo-PVI) and radiofrequency (RF) ablation with this mapping technology. Safety and feasibility data, as well as outcome information, were gathered using this elaborated ablation approach.

## 2. Methods

### 2.1. Study Participants

All the patients with symptomatic PsAF referred to our center were screened for eligibility in our study. The inclusion criteria were symptomatic PsAF and signed informed patient consent. PsAF was defined as continuously sustained AF beyond seven days, including episodes terminated by cardioversion (drugs or electrical cardioversion) after seven days.

The exclusion criteria were pregnancy, age less than 18 years old, a manifest psychiatric condition, and AF of undetermined duration. We prospectively enrolled 42 patients with PsAF, despite AAD treatment or unwillingness, to take antiarrhythmic medication. All the patients provided written informed consent and the study protocol was approved by the local ethics committee of the University of Gießen (file number 250/13).

### 2.2. Endpoints

The primary clinical endpoint of this study was termination of AF during the ablation procedure. The secondary endpoint was freedom from AF recurrence (>30 s duration) during a 12-month follow-up, with the first three months considered as a blanking period.

### 2.3. Preprocedural Management

Patients underwent transesophageal echocardiography to exclude left atrial thrombus formation. Non-vitamin K antagonist oral anticoagulants (NOAK) were withheld 24 h prior to ablation and vitamin K antagonists continued with INR between 2.0 until 2.5.

### 2.4. Noninvasive Mapping of Rotational Activity

**Figure 1:**
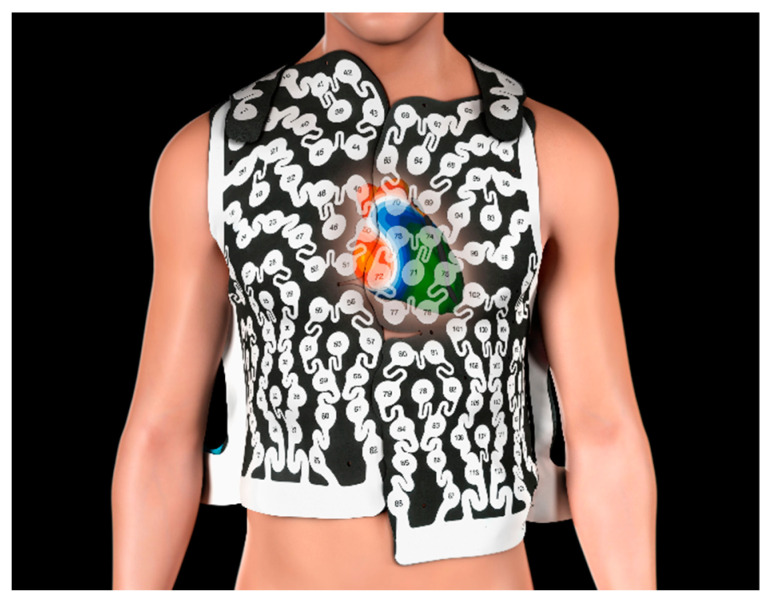
Vest with 256 electrodes. CardioInsight, Medtronic, Minneapolis, MN, USA, used with the permission of Medtronic. © 13 February 2024 Medtronic.

A noninvasive electrocardiographic mapping with a vest with 252 electrodes (CardioInsight, Medtronic, Minneapolis, MN, USA, Figure 1) in combination with low-dose cardiac computed tomography (CT) scan was performed prior to the ablation procedure to identify rotational activity with a mathematical model and project them on the surface of the atrial walls. The details of the noninvasive mapping technique have been described previously [12]. Briefly, all the patients underwent a low-dose cardiac CT scan prior to the ablation procedure, in most cases on the same day. The vest was placed on the thorax of the patient and connected to the CardioInsight mapping system in order to record unipolar surface potentials. These potentials represent the epicardial activity. To be able to map the AF without the potential disturbance of ventricular activity, adenosine was given as an intravenous bolus to create pauses with atrial activity only. If the patient was in sinus rhythm (SR) at the beginning of the ablation procedure, AF was induced by atrial high rate stimulation. Care was taken to start the first phase map analysis after persistence of the AF for at least 30 min. The mapping system reconstructed the different segments with unipolar potentials and projected them on the surface of the atrial walls. Signal filtering and phase mapping was combined to create maps of the right and left atrium as well as the septum. Rotational activity was considered to be significant if one wave rotated more than 1.5 times around a center on the phase map (in an area < 2 cm^2^), as described previously [16]. The mapping was repeated after completion of the pulmonary vein isolation.

The spatial resolution of the noninvasive mapping system has been described earlier and the determination of the true site of origin of focal activity is possible with sufficient accuracy with consideration of error estimates of up to 20 mm in a porcine model [17].

### 2.5. Ablation Procedure

**Figure 2:**
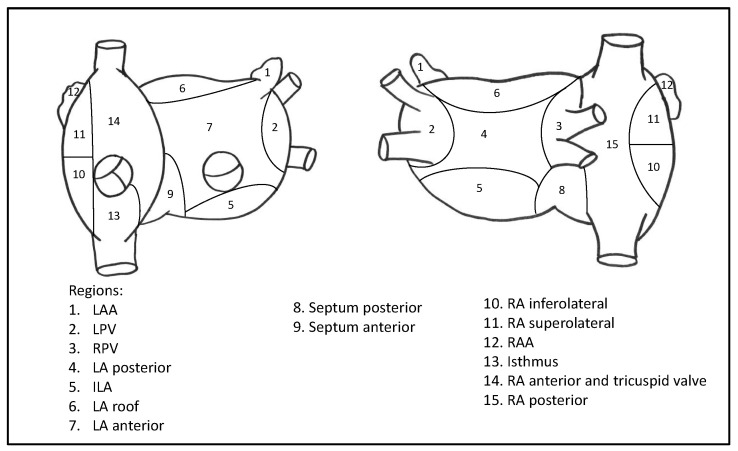
Right and left atrium in anterior (**left**) and posterior (**right**) views. Abbreviations: LAA = left atrial appendage, LPV = left pulmonary veins, RPV = right pulmonary veins, LA = left atrium, ILA = inferior left atrium, RA = right atrium, RAA = right atrial appendage.

Most of the ablation procedures were performed in deep sedation with analgesia, but in selected patients general anesthesia was used, due to comorbidities or patient request. After transseptal puncture, intravenous heparin was infused to achieve a targeted activated clotting time of 300 s. Angiography of the left atrium and the pulmonary veins was performed. In patients with SR before ablation, AF was induced by atrial high rate pacing. The following areas were distinguished in the left and right atrium: left atrial appendage (LAA), left pulmonary veins (LPV), ridge between the LAA and the LPV, right pulmonary veins (RPV), posterior left atrium, inferior left atrium (ILA), left atrial roof, anterior left atrium, posterior septum, anterior septum, inferolateral right atrium, superolateral right atrium, right atrial appendage (RAA), right atrial isthmus, right anterior region and tricuspid valve, and posterior right atrium (see Figure 2 for details).

For cryo-PVI, a 28-mm fourth generation cryoballoon (Arctic Front Cardiac Advance Pro Cryoablation Catheter, Medtronic, Minneapolis, MN, USA) was advanced through a steerable 15-F sheath. Details of the PVI procedure with the cryoballoon have been described previously [18]. In brief, the balloon was placed at the antrum of each pulmonary vein, occlusion was confirmed by the injection of a contrast agent, and one or more cryoablations were performed until the pulmonary veins were successfully isolated. During the ablation of the right pulmonary veins, the phrenic nerve function was monitored with compound motor action potential (CMAP).

After the successful cryoablation of the pulmonary veins, an electroanatomic map of the left and right atrium was created with a 3D-mapping system (EnSite Precision^TM^, Abbott, Chicago, IL, USA) and a 10-pole lasso catheter (Advisor^TM^ FL Circular Mapping catheter, Abbott, Chicago, IL, USA). In patients with SR after PVI (2/42), AF was reinduced with atrial high rate pacing. The rotational activity was analyzed again and then targeted by contact force-controlled, open-irrigated RF catheter ablation (Tacticath™ SE™, Abbott, Chicago, IL, USA). The RF energy was delivered with a power of 25 to 35 W (higher energy at the anterior wall and lower at the posterior wall). If there was conversion of the AF into atrial tachycardia (AT) during ablation of rotational activity, the AT was mapped and, if feasible, ablated until termination into SR. If the AF persisted at the end of the ablation procedure, the patients were cardioverted into SR (either by flecainide injection or by electrical cardioversion). At the end of the procedure, the status of PVI was assessed again in SR with the 10-pole lasso catheter (entrance and exit block). In cases where reconduction occurred, the pulmonary vein was targeted with RF energy until complete and persistent isolation was achieved.

### 2.6. Follow-Up

The patients were followed at our outpatient unit 3, 6, 9, and 12 months after the procedure and screened for arrhythmia recurrence (history, ECG, 7-day Holter). Moreover, the patients were encouraged to keep a personal rhythm log and see a doctor if they experienced arrhythmia-related symptoms in order to have a 12-channel ECG recorded. During the 3- and 12-month follow-up assessments, echocardiography was performed. If there was arrhythmia recurrence (>30 s of documented AF or atrial tachycardia) after the 3-month blanking period, the patient was offered a redo-procedure.

### 2.7. Statistical Analysis

Categorical data are presented as absolute numbers and percentages and continuous data as mean ± standard deviation or as median with interquartile range [first and third quartile]. The data were compared using Student’s *t*-test, Mann—Whitney U-test, or ANOVA with pre-tests. A log-rank (Mantel—Cox) test was performed to compare the outcome between groups, depending on the peri-interventional result. Two-sided P-values less than 0.05 were considered to reject the null hypothesis. The statistical computations were performed with Graphpad Prism 8 for macOS (version 8.4.0; GraphPad Software, San Diego, CA, USA).

## 3. Results

From October 2017 to May 2020, 42 patients were included in the study. The mean follow-up period was 13 ± 2.8 months, the mean patient age was 65 ± 8 years, and 64% were male (Table 1). The median uninterrupted AF duration was four months (interquartile range 2–11 months). The patients had a mean left atrial area of 21.6 ± 5 cm^2^ and a mean right atrial area of 19.6 ± 4 cm^2^ with a normal mean left ventricular ejection fraction of 58.4% ± 6.42%. Somewhat less than half (42.9%) of the patients were on AAD prior to the ablation procedure. The prevalence of comorbidities was rather low, with a mean CHA_2_DS_2_-VASc score of 2.1 points. The patients had normal renal function (GFR 90.1 ± 24.1 mL/min) and normal body mass index (28.5 ± 4 kg/m^2^). At the start of the ablation procedure, 19% of the patients were in sinus rhythm and required high rate atrial pacing to induce atrial fibrillation and enable phase mapping for rotational activity.

The mean total procedural time was 274 ± 48 min and the fluoroscopic time was 27 ± 7 min (Table 2). The total RF ablation procedure duration was more than two times longer than the cryoablation procedural time (185 ± 27 min vs. 88 ± 23 min). The amount of contrast agent used was 29.8 ± 8.3 mL. Noninvasive electrocardiographic mapping with the 252-electrode vest identified rotational activity in every patient of our cohort. The mean count of the total biatrial rotational activity was 5 ± 1.3, and the rotational activity was more common in the left atrium than in the right atrium (3.5 ± 0.9 vs. 1.5 ± 0.8; *p* < 0.001).

Cryo-PVI was successful in all patients, and two patients converted to SR during PVI. The location and amount of rotational activity before and after PVI did not show any differences, apart from rotational activity inside the pulmonary veins and the anterior right atrium/tricuspid valve (see Table 3). In the majority of patients, rotational activity was identified in both atria. The locations with the most common rotational activity were (Table 3): posteroseptal (37/42), inferior left atrium (36/42), posterior left atrium (34/42), anteroseptal (31/42), the ridge between the left atrial appendage and the left superior pulmonary vein (25/41), the right atrial appendage (23/42), the superolateral right atrium (17/42), and the inferolateral right atrium (14/21). Only a few patients showed rotational activity at the left atrial roof (5/42).

Ablation of the rotational activity in the left atrium was performed in 41 patients (97.6%) and in the right atrium in 33 patients (78.6%). Periprocedural complications were rather rare, despite the extensive ablation strategy. There was one case of pericardial tamponade managed by percutaneous pericardiocentesis, which led to early termination of the procedure after cryo-PVI without further targeting of rotational activity. One patient developed periprocedural hemoptysis, which led to the termination of the ablation procedure after cryo-PVI and complete ablation of left atrial rotational activity. Another patient needed transvenous stimulation due to sinus arrest, which resolved after postprocedural discontinuation of antiarrhythmic drugs. No patient died during the follow-up period.

**Figure 3:**
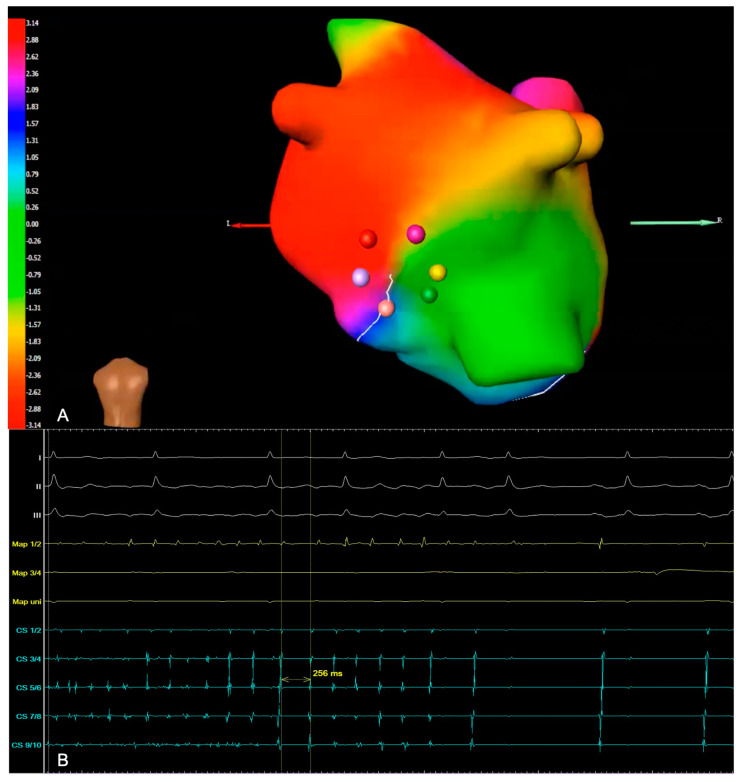
(**A**) Example of a phase map with rotational activity, with a view of the inferior left atrium. It shows a wavefront of the rotor, with the colors representing the activation sequence. Ablation in this area terminated the AF in this particular patient. (**B**) Intracardiac electrogram (EGM) illustrating periprocedural termination of AF and conversion to sinus rhythm (Map: ablation catheter; CS: coronary sinus).

**Figure 4:**
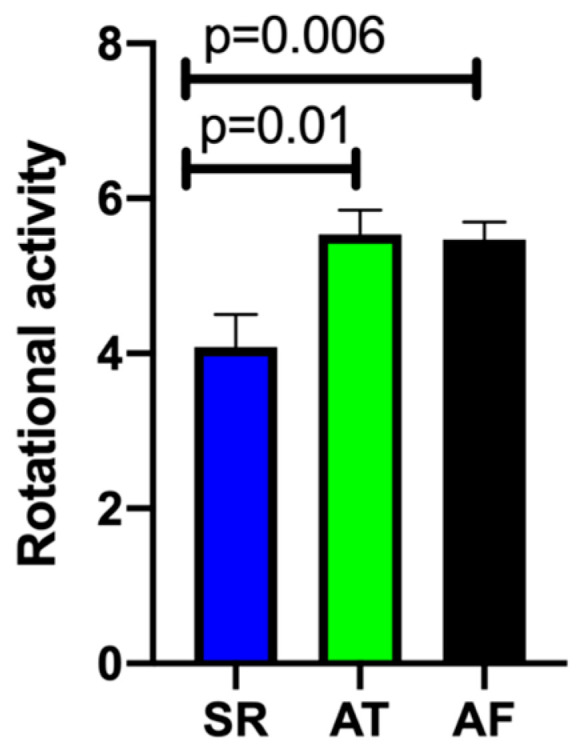
Total number of sites with localized rotational activity. Blue column: Group A—Termination of atrial fibrillation (AF) to sinus rhythm (SR); Green column: Group B—Conversion from AF to atrial tachycardia (AT); Black column: Group C—Ongoing AF until cardioversion at end of procedure.

A total of 25 patients (59.5%) reached the primary endpoint of periprocedural termination of AF (Table 4). During ablation (cryo-PVI or ablation of rotational activity), 12 patients converted from AF directly to SR (28.6%; Group A). Another 13 patients converted from AF to AT (30.9%; Group B). One example of a phase map with rotational activity in the inferior left atrium (ILA) where ablation terminated the AF is shown in Figure 3. In 17 patients, AF continued until the end of the procedure and electrical cardioversion was performed (40.5%; Group C). A lower amount of rotational activity seemed to be amenable to converting AF under ablation to SR, as illustrated in Figure 4. No difference was found between Groups B and C in the number of rotors detected (Group A 4.08 ± 0.42; Group B 5.54 ± 0.31; Group C 5.47 ± 0.23; *p* = 0.01 A vs. B and *p* = 0.006 A vs. C). Table 5 depicts the areas with AF termination during RF ablation: AF termination occurred more often during ablation in the left atrium.

After the three-month follow-up, 86% of patients were free from any atrial arrhythmia. No significant differences were observed between groups (A: 91.7%; B 92.3%; C: 76.5%; *p* = 0.38).

**Figure 5:**
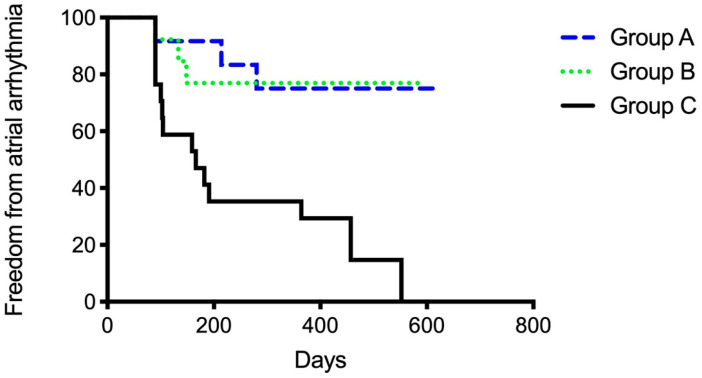
Secondary endpoint of freedom from any atrial arrhythmia grouped according to the primary endpoint of periprocedural ablation result. Group A: Termination of atrial fibrillation (AF) to sinus rhythm. Group B: Conversion from AF to atrial tachycardia (AT). Group C: Ongoing AF until cardioversion at end of procedure.

**Figure 6:**
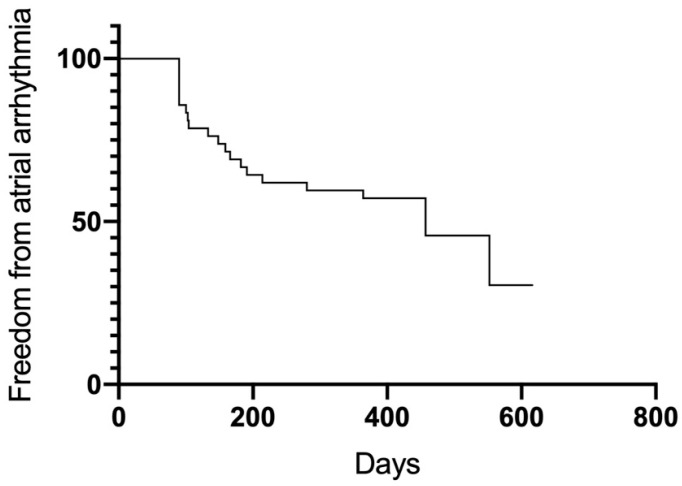
Freedom from any atrial arrhythmia.

**Figure 7:**
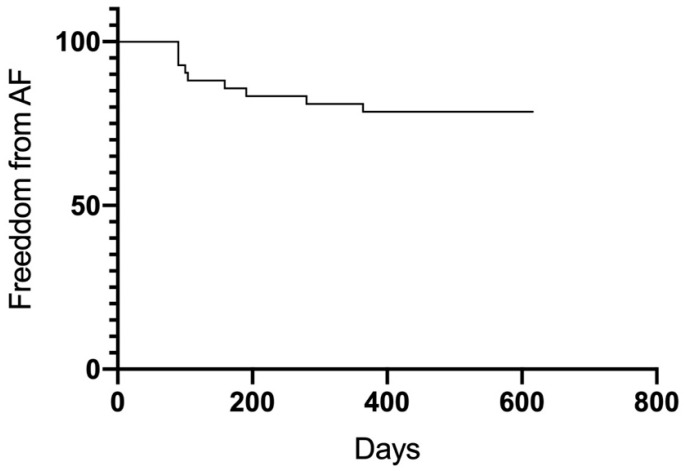
Freedom from atrial fibrillation.

After the 12-month follow-up, a total of 23 patients were free from AF and AT (54.8%). Significant differences in outcome were observed between groups (Figure 5). Groups A (75%) and B (77%) had higher success rates than Group C (17.6%) (A vs. C *p* = 0.005; B vs. C 0.007). Between Group A and B, no significant differences were observed (*p* = 0.98). Thirty-four patients overall (81%) were free from AF recurrence. Nineteen patients (45.2%) had a recurrence of atrial arrhythmias, and the majority of them (12 patients, 28.6%) developed AT in the course of the follow-up period (Figure 6 and Figure 7). Among the patients with peri-interventional conversion from AF to SR (Group A) or AF to AT (Group B), only one patient in each group had a recurrence of AF (8.3% vs. 7.7%, respectively).

Eight patients underwent a second ablation procedure. Notably, among the 19 patients with recurrences of atrial arrhythmias, only five patients developed arrhythmias of a persistent character (11.9%). After completion of the follow-up, the proportion of patients on AADs (16.7%) was reduced to a third of that prior to the ablation procedure. In the course of follow-up, three patients (7.1%) needed implantation of a permanent dual-chamber pacemaker, two cases due to sick sinus syndrome and one case due to AV block II° type I.

## 4. Discussion

To the best of our knowledge, the present Cryo-Vest Study is the first prospective study to assess the safety and efficacy of biatrial rotor ablation combined with cryo-PVI in patients with PsAF. Moreover, it is the first time a structured analysis of potential changes in rotational activity after cryo-PVI was performed.

The study cohort can be described as rather healthy, based on a low CHA_2_DS_2_-VASc score, good renal function, and only moderately dilated left atrium. As is typical for a cohort with PsAF, the majority of patients were male. A median of four months of uninterrupted AF underlines the tenacity of the arrhythmia in our patient cohort. The time from the first diagnosis of AF to the ablation procedure, with a median of 33 months, was rather long, especially if one takes into account the knowledge that long-term rhythm control is more difficult to achieve by ablation when the time from first AF diagnosis is prolonged [19,20], with success rates declining with each year after diagnosis.

Three patients required pacemaker implantation after the ablation procedure, which is more than can usually be expected in daily routine. One patient had a long AV block (PR-interval of 310 ms) prior to ablation, and required pacemaker implantation to allow antiarrhythmic therapy because of recurrent episodes of atrial tachycardia despite ablation and reablation. The second and third patients developed a symptomatic sick sinus syndrome shortly after the ablation procedure. In these patients, no ablations were performed close to the sinus node, but directly inside the right atrial appendage with a large atrial signal. One of these patients had long-standing persistent atrial fibrillation, and it is well known that the co-occurrence of atrial fibrillation and sick sinus syndrome is quite high in this population [21]. However, because we performed the ablation while the patients were in AF, we could not monitor for adverse signs of sinus node modification as sinus bradycardia, sinus arrest, or sinus tachycardia. According to the study protocol, we deliberately ablated in AF to be able to analyze the site of AF termination. In conclusion, we do not consider the pacemaker implantations in these three patients to be a direct complication of the ablation procedures, rather than of the patients’ diseased atrium, with concomitant conduction delay.

The total procedural time can be regarded as excessive, making this approach physically demanding for the patient as well as for the treating electrophysiologist. The main cause for the long duration is the RF ablation of rotational activity, which required more than twice the time needed for cryo-PVI. To our surprise, the location and amount of rotational activity before and after PVI did not show any differences, apart from a decline in rotational activity inside the pulmonary veins and the right anterior atrium/tricuspid valve. On the one hand, this can be interpreted as a rather positive intraprocedural validation process of the mapping technique, but, on the other hand, it raises the question how many patients with PsAF worldwide are successfully treated by PVI only. The fact that PVI led to a decline in rotational activity in the right anterior atrium/tricuspid valve makes no sense at first glance, but taking into consideration the analysis of 12 extra-PV regions, the statistical deviation of only one region after PVI can be attributed to either statistical imprecision due to the sample size or methodological/technical imprecision.

Furthermore, it can be debated which rotational activity is of clinical interest at all. In previous studies such as the AFACART study [16], the drivers of AF were considered significant when wavefronts rotated more than 1.5 times within a spatially stable area (<2 cm^2^), which was an assumption adopted in our study. The results concerning the localization of the rotational activity were comparable to those of the present study, as was the periprocedural success of termination of AF.

Rotational activity was found in every patient, whereby rotational activity was more common in the left than in the right atrium. Notably, recent studies have indicated that patients with ongoing AF, after completion of a predetermined left atrial ablation strategy, exhibit sources at less common anatomical sites such as the right atrium, and in these particular patients further right atrial ablation strategy resulted in greater success of AF termination and less recurrence of atrial arrhythmias when compared with those without termination [22,23,24].

Interestingly, the majority of our patients displayed rotational activity in the posteoseptal and posterior left atrium, which can be eliminated by the empirical creation of a posterior box lesion, a common approach in many hospitals. In contrast, the creation of a left atrial posterior wall box does not seem to routinely confer any further benefit to patients with PsAF, as revealed by data from the CAPLA trial [25,26,27]. In particular, a posteroseptal driver might remain unaffected by the creation of a posterior box lesion. One could argue that a “one size fits all” ablation concept is not suitable for patients with PsAF, but it also should be noted that even a personalized fibrosis-guided concept plus conventional PVI showed no difference in arrhythmia recurrence compared with conventional PVI in the DECAAF II trial [28]. In fact, additional ablation targets in the “PVI plus” group were associated with an increased risk of peri-interventional complications. This might indicate that not every sort of scar plays an active role in electrical triggering and/or perpetuation of AF.

The lack of a clear path forward in treating PsAF is why we investigated a technique that relies purely on electrical atrial activity and delivers a personalized result. Since a single approach to ablation of rotational activity in patients with paroxysmal AF showed negative results compared with PVI [29], we sought to combine both approaches in our patient cohort with PsAF. Nevertheless, current survey data on strategies for AF ablation in patients with PsAF reveal that 88% of electrophysiology centers perform PVI only, 2% choose PVI plus linear ablation, and 10% choose PVI plus defragmentation and/or substrate modification [30].

The majority of our patients (59.5%) reached the primary endpoint of periprocedural termination of AF: 28.6% converted directly from AF to SR; and 30.9% converted from AF to AT. This termination rate is high compared with that of classic PVI as a stand-alone procedure. The termination rates in our study were very similar to those of Kis et al. and Knecht et al.; study groups that used the same noninvasive mapping strategy (58% and 63% periprocedural termination of AF; respectively) [16,31].

The clinical impact of termination of AF as a procedural endpoint during ablation in PsAF still seems to be ambiguous. One major, prospective multicenter clinical trial was undertaken by Elayi and colleagues when investigating a cohort of 306 patients over a mean follow-up period of 25 months. Unlike our study, the ablation strategy consisted of pulmonary vein antrum isolation followed by CFAE ablation. With the advent of new panoramic mapping techniques, re-entrant and focal sources driving persistent AF were expected to be identified with more specificity when compared with CFAE [32]. This study group was also able to achieve peri-interventional AF termination in 58% of all patients, but during follow-up time there was no difference in rhythm control compared with the patients without periprocedural termination of AF [33]. In a meta-analysis of 20 studies that directly compared the clinical outcome of patients in whom AF termination was achieved during ablation with those in whom it was not, the majority of studies (17 of 20) demonstrated significantly superior outcomes in the group with procedural AF termination [34]. In addition, a study by Rostock et al. that assessed the long-term predictors of single- and multiple-procedure outcomes for patients with PsAF, AF termination during the index procedure significantly predicted single-procedure success and also a favorable outcome after the final procedure [35]. While 45.2% of patients with periprocedural AF termination showed a recurrence of atrial arrhythmias, the majority of them (28.6% of the total) developed AT in the course of the follow-up period. This is in line with findings from other groups investigating postinterventional follow-up of PsAF patient cohorts [36]. Although ablation of AT can sometimes be challenging, especially in epicardial localized macroreentry tachycardias, overall AT can be targeted with a high interventional success rate of up to 90% and can be often be considered as the last stage in achieving long-term rhythm control [37,38,39]. The fact that in our study among the patients with peri-interventional conversion from AF to SR (Group A) or AF to AT (Group B) only one patient in each group developed recurrence of AF heavily underlines the predictive value of peri-interventional success in long-term maintenance of SR.

International electrophysiologists have been on a quest for the past two decades to discover ablation therapies in addition to PVI that might substantially increase successful rhythm control in patients with PsAF. While a number of promising results had been observed in non-randomized, smaller trials, once these innovative ablation strategies were subjected to larger, multicenter randomized trials, no additional benefit in rhythm control was documented. Our study contributes another piece to the puzzle, by demonstrating the maintenance of SR in patients with PsAF. Due to the relatively long time needed to carry out this extensive procedure, however, and its requirement for additional human resources for technical support, including prior CT, it would not appear to be transferable to the clinical routine as a regular first approach in patients with PsAF. The application of this technique might be considered in a second (or more) ablation procedure; however, further studies are necessary to confirm whether the results may be applied to redo procedures, since all data were collected from ablation-naïve patients. Furthermore, the procedure time could be significantly reduced by new ablation techniques, such as pulsed-field ablation (PFA). PFA has recently been evaluated in patients with PsAF as a nonthermal method for ablating myocardial tissue more selectively. In the study by Reddy et al., PFA was used for PVI as well as for posterior wall isolation, and the latter was performed in less than 10 min [40]. An interesting approach would be to combine the advantages of rotor-guided mapping (as described in our study) with the more tissue-selective and rapid ablation technique of PFA in future studies.

## 5. Limitations

This is a single-center study. Given the limited size of the patient population, the cohort may be heterogeneous. A subgroup analysis according to the CHA2DS2-VASc score is lacking. The sample size group was empirical and not calculated.

A general technical aspect of this noninvasive electrocardiographic mapping of atrial rotational activity is the epicardial projection on the atrial walls. All 3D ablation systems are based on endocardial electroanatomic mapping. A slight anatomic difference in the anatomic maps cannot be confirmed with absolute certainty, especially since the maps were not graphically embedded in one another. Until now, there exists no 3D ablation platform that allows the integration of the vest’s map. Another technical challenge lies within the interpretation of electrical signals of the atrial septum, which cannot be projected epicardially. Moreover, despite the thinner atrial myocardium, compared to the ventricle, it can be sometimes very demanding to ablate atrial epicardial structures, especially when there is no certain feedback of successful ablation beyond loss of endocardial capture and termination of atrial fibrillation. Also, the choice of definition of rotational activity is rather empirical.

## Figures and Tables

**Table 1:** Baseline characteristics.

Age, Years	65 ± 8
Sex, male n (%)	27 (64)
Uninterrupted AF duration (months)	4 (2–11)
Time from first diagnosis of AF to ablation, months	33.8 ± 46.1
Left atrial area, cm^2^	21.6 ± 5
Right atrial area, cm^2^	19.6 ± 4
Antiarrhythmic drugs (class I and III) prior to procedure n (%)	18 (42.9)
LVEF, %	58.4 ± 6
GFR, mL/min/1.72 m^2^	90.1 ± 24.1
BMI, kg/m^2^	28.5 ± 4
CHA2DS2Vasc score n (%)	
0–1	16 (38)
2–3	18 (43)
>3	8 (19)
Hypertension n (%)	33 (78.5)
History of stroke n (%)	2 (4.8)
Diabetes n (%)	6 (14.3)
Number of prior cardioversions	1 (0–3)
SR at start of procedure n (%)	8 (19)

Values represent n (%) or mean ± standard deviation or median (interquartile range, IQR). Abbreviations: AF, atrial fibrillation; BMI, body mass index; GFR, glomerular filtration rate; LVEF, left ventricular ejection fraction; SR, sinus rhythm.

**Table 2:** Procedural characteristics.

Total procedure time, min	274 ± 48
Procedure time cryo-PVI, min	88 ± 23
Procedure time RF ablation, min	185 ± 46
Cryo application time, min	17.4 ± 5.1
Total RF ablation time, min	61.2 ± 23.6
Total RF ablation energy, Ws	103,485 ± 38,333
Total fluoroscopic time, min	27 ± 7
Total dose-area product, Gycm^2^	10.8 ± 15.8
Contrast agent, mL	29.8 ± 8.3
Total number of rotors	5 ± 1.3
Rotors in left atrium	3.5 ± 0.9
Rotors in right atrium	1.5 ± 0.8

Values represent mean ± standard deviation. Abbreviations: PVI, pulmonary vein isolation; RF, radiofrequency.

**Table 3:** Number of rotors before and after PVI in the designated areas of the left and right atrium.

Area	Before PVI		After PVI		*p*-Value
	(*n* = 41)		(*n* = 41)		
LAA	25	60.98%	22	53.66%	0.629
LAA-LPV	24	58.54%	25	60.98%	1.000
LPV	6	14.63%	0	0.00%	0.031
RPV	3	7.32%	0	0.00%	0.250
Posterior LA	31	75.61%	34	82.93%	0.549
ILA	39	95.12%	36	87.80%	0.453
LA Roof	4	9.76%	5	12.20%	1.000
LA anterior	18	43.90%	19	46.34%	1.000
Posteroseptal	39	95.12%	37	90.24%	0.687
Anteroseptal	35	85.37%	31	75.61%	0.344
Inferolateral RA	19	46.34%	14	34.15%	0.332
Superlateral RA	19	46.34%	17	41.46%	0.791
RAA	22	53.66%	23	56.10%	1.000
RA isthmus	13	31.71%	11	26.83%	0.754
Anterior right atrium/tricuspid valve	12	29.27%	3	7.32%	0.012

Values represent n and %. Abbreviations: LAA, left atrial appendage; LPV, left pulmonary veins; RPV, right pulmonary veins; LA, left atrium; ILA, inferior left atrium; RA, right atrium; RAA, right atrial appendage.

**Table 4:** Outcome characteristics.

Direct conversion from AF to SR under ablation of rotational activity	12/42 (28.6%)
Conversion from AF to AT under ablation of rotational activity	13/42 (31.0%)
Ongoing AF until external electrical cardioversion at the end of procedure	17/42 (40.5%)

Abbreviations; AF, atrial fibrillation; AT, atrial tachycardia; SR, sinus rhythm.

**Table 5:** Areas where atrial fibrillation terminated with conversion to either sinus rhythm or atrial tachycardia.

Region	Number of Patients
RA lateral	2
RA posterior	1
RAA	2
RA inferolateral	2
RA anterior TV	1
Posteroseptal	1
LA posterior	3
ILA	4
LA anterior	1
Anteroseptal	2
LA septal	1
LAA	1
LSPV	1
RSPV	1

For definition of the areas see Figure 2. Abbreviations: RA, right atrium; RAA, right atrial appendage; LA, left atrium; ILA, inferior left atrium; LAA, left atrial appendage; LSPV, left superior pulmonary vein; RSPV, right superior pulmonary vein; TV, tricuspid valve.

## Data Availability

The data presented in this study are available on request from the corresponding author.
